# Identification of Tartrazine adulteration and evaluating exposure to synthetic dyes of sunset yellow and Quinoline yellow through consumption of food products among children

**DOI:** 10.1002/fsn3.2975

**Published:** 2022-07-30

**Authors:** Hannaneh Rahnama, Seyed Mohammad Mazloomi, Enayat Berizi, Azam Abbasi, Zahra Gholami

**Affiliations:** ^1^ Nutrition Research Center, Department of Food Hygiene and Quality Control, School of Nutrition and Food Sciences Shiraz University of Medical Sciences Shiraz Iran; ^2^ Food Control Laboratory, Department of Food and Drug Shiraz University of Medical Science Shiraz Iran

**Keywords:** quinoline yellow, sunset yellow, synthetic dye, tartrazine adulteration

## Abstract

Excessive consumption of synthetic food dyes by children may raise concerns about their health. These dyes may aggravate the hyperactivity symptoms and exacerbate asthma in sensitive children. The purpose of this study was to determine the presence of sunset yellow and quinoline yellow dyes, as well as tartrazine in dairy‐free fruit ice cream, freeze pop, jelly, and candy. Additionally, we evaluated the amount of two food dyes consumed by children. To do so, a total of 150 food samples, including 20 dairy‐free fruit ice creams, 25 freeze pops, 57 jelly products, and 48 types of candy were randomly selected from stores in Shiraz, Iran. Then, using the high‐performance liquid chromatography (HPLC) method and an ultraviolet (UV) detector, we measured the amounts of sunset yellow and quinoline yellow dyes and identified the use of tartrazine. Also, the per capita consumption (grams per day) of the mentioned foods was calculated using a checklist in two groups of male and female primary schoolchildren aged 6–9 years and 10–13 years in Shiraz, Iran. According to the results, 11 (7.33%) samples contained only tartrazine and 107 (71.33%) samples contained quinoline yellow and sunset yellow synthetic dyes. In addition, of 107 samples that used quinoline yellow and sunset yellow, 102 (95.33%) contained unauthorized tartrazine. Only seven (6.54%) samples contained exceedingly high concentrations of authorized quinoline yellow and sunset yellow synthetic dyes. However, the exposure assessment showed that the intake of quinoline yellow and sunset yellow was at average levels and the 95th percentile in both age groups was less than the associated acceptable daily intake (ADI). For synthetic dyes, the target hazard quotient (THQ) and hazard index (HI) were less than one, indicating that ingestion of these two dyes via food products does not pose a risk to children's overall health.

## Graphical abstract

The purpose of this study was to determine the presence of sunset yellow and quinoline yellow dyes, as well as tartrazine in dairy‐free fruit ice cream, freeze pop, jelly, and candy. A total of 150 food samples, including 20 dairy‐free fruit ice creams, 25 freeze pops, 57 jelly products, and 48 types of candy were randomly selected from stores in Shiraz, Iran. For synthetic dyes, the target hazard quotient (THQ) and hazard index (HI) were less than one, indicating that ingestion of these two dyes via food products does not pose a risk to children’s overall health.

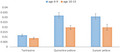

## 1 INTRODUCTION

Recently, there has been a surge of interest in the issue of food additives, particularly food dyes. Today, synthetic dyes are widely used in a variety of foods, including snacks, beverages, ice cream, and others, due to their resistance to light, temperature, and oxidation, which can enhance the attractiveness of such products (Qi et al., 2015; Rovina et al., 2016). In this regard, it has been shown that the decision to purchase a particular product is heavily influenced by its color. Additionally, children prefer warm and vibrant colors such as orange, yellow, and red (Hunjet & Vuk, 2017).

Synthetic food dyes, like drugs and contaminants, are biologically incompatible with the human body, and some of these dyes may be metabolized by enzymes and absorbed or excreted following consumption (Kuno & Mizutani, 2005). The metabolized dyes may become toxic and pose a health risk to the consumer, particularly if they are consumed in large quantities (Vachirapatama et al., 2008; Rezaei et al., 2015; Gholami et al., 2021). Some studies have shown that dyes such as sunset yellow and tartrazine can cause allergic reactions such as dermatitis, asthma exacerbation in sensitive individuals, urticaria, and restlessness; they also exacerbate sleep disorders and hyperactivity in children. Moreover, research has shown the mutagenic effect of quinoline yellow at concentrations above the standard (the maximum amount of permitted quinoline yellow is 200 μg/mL) in human lymphocytes (EFSA, 2009; Chequer et al., 2015). Additionally, research suggests that color additives may harm the behavioral responses of sensitive children (Stevens et al., 2013). Numerous studies have reported the widespread and unregulated use of such dyes in children's foods. For example, studies conducted in Thailand (2008) and India (2010) found that authorized synthetic dyes were consumed higher than the standard amounts, and in some cases, unauthorized dyes were also used in foods (S Dixit et al., 2010; Vachirapatama et al., 2008). Several studies in Iran have reported the use of unauthorized concentrations of artificial food dyes, as well as the use of unauthorized artificial colors in food products (Farzianpour et al., 2013; Sadeghi et al., 2020). So, it is essential to control the foods containing synthetic dyes.

In general, in many countries, the type of artificial food dyes and the permitted concentrations are regulated by national standards (INSO, 2013; EFSA, 2009; World Health Organization, 2011). In Brazil, for example, international regulations restrict the concentration and use of several different food dyes, allowing only eight synthetic colors. While 15 synthetic colors are permitted in the European Union, only nine synthetic colors are permitted in the United States (Alves et al., 2008; Ha et al., 2013; Oplatowska‐Stachowiak & Elliott, 2016). Iran permits the use of only seven synthetic food dyes, including sunset yellow and quinoline yellow; however, tartrazine is an unauthorized dye in Iran. But in the international codex standard, all three colors of sunset yellow, quinoline yellow, and tartrazine are allowed artificial colors (INSO, 2013; IACM, 2020).

In Iran, there is no standard for the maximum concentration of sunset yellow and quinoline yellow in some foods such as candy and jelly, which may cause excessive use of these colors. Children find yellow and orange to be the most appealing colors, but excessive consumption of food dyes can endanger public health. There have been few studies conducted to determine the extent to which children are exposed to such colors in Iran.

Accordingly, this study aimed to measure the concentration of sunset yellow and quinoline yellow dyes in four types of food consumed by children and the exposure of primary schoolchildren in Shiraz, Iran to these two colors. We also assessed the non‐carcinogenic health risks of these two dyes and identified the presence of unauthorized tartrazine dye in the foods consumed by children.

## 2 MATERIALS AND METHODS

### 2.1 Sampling and design

For food sampling, Shiraz city was divided into four geographical regions. In each region, 10 stores were randomly selected. Then, the samples from all brands with yellow and orange colors were collected from November to December 2016. In this study, 150 food samples including 20 dairy‐free fruit ice cream, 25 freeze pops, 57 jelly products, and 48 types of candy were collected and the dyes contained in them were determined using the high‐performance liquid chromatography (HPLC) method and an ultraviolet (UV) detector. To determine the extent of exposure, 402 primary schoolchildren aged 6–13 years from both genders were studied. The participants were the children of the staff of Shiraz University of Medical Sciences (SUMS). The study protocol was reviewed and approved by the SUMS (ethical code: IR.SUMS.REC.1399.760). The staff that agreed to participate in the study completed the electronic checklists for their children.

Using the information obtained from the checklist, the amount of food products consumed by the children in a week was calculated for each person. Then, considering the weekly consumption of each food product, the daily consumption rate was estimated for each person according to formula (1).

Formula 1:

The consumption rate of each food product separately for each individual (grams per day) = The weekly consumption rate of each product separately (grams per week)/number of the days of a week (7 days).

Then, the concentration of each artificial food dye in each product was calculated separately and the amount of chronic daily intake (CDI) was obtained for sunset yellow and quinoline yellow dyes. Then, we calculated the average daily intake of each dye for each individual separately. Finally, we determined the average intake of each dye for each age group. Additionally, the highest average consumption of each food was calculated for both age groups.

### 2.2 Equipment and reagents

We employed the Alliance HPLC System equipped with a two‐channel pump, a UV detector, and a degasser. The isolations were performed on an 18‐C chromatographic column (4.6 mm 250 mm, 5 m), and the wavelengths of each color were determined using a Dynamica Halo DB‐20R UV–VIS Spectrophotometer. We purchased the standard dyes sunset yellow (E110) and quinoline yellow (E104) and tartrazine (E102) from the Sigma‐Aldrich company (Germany). Also, Acetonitrile grade for HPLC, methanol, concentrated acetic acid, and 25% ammonium hydroxide were purchased from the Merck Company (Germany).

The calibration curve was constructed using a standard mixture of three dyes: tartrazine, sunset yellow, and quinoline yellow. The standard mixture was prepared in five concentrations of 0.75, 1.25, 2.5, 5, and 10 mg/L and stored at 4°C. We used the modified isocratic method with mobile phase including acetonitrile (190 ml), methanol (10 ml), concentrated acetic acid (5600 μl), and 25% ammonium hydroxide (8300 μl). The solution volume was increased to 1000 by adding deionized water, and finally the pH was adjusted to 5.95 by adding NH4OH. The injection volume of the sample was 20 μl, and the column temperature was kept constant with the ambient temperature. The mobile phase flow rate was set to 0.8 ml/min. Peaks were detected in two channels with wavelengths of 482 nm and 420 nm using a UV detector. Tartrazine and quinoline yellow were read at 420 nm in channel one, while sunset yellow was read at 482 nm in channel two. The colors were classified according to their retention time.

### 2.3 Analytical quality assurance

To confirm the method for qualitative and quantitative measurement of artificial food colors in food products, we used different validation parameters, including calibration curve, retention time, correlation parameter, relative standard deviation (% RSD), limit of detection (LOD), and limit of quantitation (LOQ). The permissible limit of each parameter was determined using the standards by the Association of Official Analytical Chemists (AOAC, 2002) in 2002. To obtain the calibration curve, standard solutions of each color were injected into the HPLC as triplicates. To evaluate the reproducibility of measurement for each permitted food dye, a sample containing each of these compounds was prepared and injected into the HPLC as triplicates. To investigate the repeatability, RSDr was calculated for the observed peak area deviations, the calculated concentration, and the retention time. The results obtained from the calibration curves, including correlation coefficient, retention time, % RSD, LOD, and LOQ are presented in Table 1.

**TABLE 1:** Quality assurance data on standard permitted and non‐permitted colors

Retention time (tR), calibration data including λ, correlation coefficient (r), linear regression, limit of detection (LOD), and limit of quantitation (LOQ) for three synthetic dyes.								
Name	Calibration data							
	t_R(min)_	λ (nm)	r	Linear regression	^b^Recovery range (%)	^c^RSD% (n = 3)	LOD (mg/kg)	LOQ (mg/kg)
Tartrazine(E102)	3.09	420	0.999	y = 54,981x + 2213.3	106.3–112.2	1.2–3	0.08	0.24
^a^Quinolin yellow(E104)	5.06 7.21	420	0.998	y = 47043x + 6313.4	84–84.8	4.2–5.9	0.09	0.28
Sunset yellowFCF(E110)	5.69	482	0.9982	y = 59528x + 5804.7	95.1–96	1.1–2.4	0.06	0.21

^a^ Quinoline yellow has two peaks at two different times 5.06 and 7.21(min).

^b^ Recovery range: minimum and maximum recovering range in the four products tested.

^c^ RSD: relative standard deviation.

### 2.4 Preparation of samples

We melted ice cream and freeze pop samples at room temperature, and jelly samples in a water bath at 90°C. Following homogenization, a 25‐ml flask was filled with 10 ml of each sample using 2‐ml ammonium acetate buffer, 50:50 deionized water, and methanol. After pulverizing the solid candy samples, 5 grams of each sample were homogenized and brought to volume in the same manner as the liquid samples. Following that, all final samples were centrifuged for 5 min at 5000 rpm and 30°C, and 20 μl of each sample was injected into HPLC after passing through a syringe filter with a diameter of 0.22 mμ. Then, the samples were tested to determine the concentration of each synthetic dye.

### 2.5 Calculating the consumption rates of foods and synthetic food dyes

CDI was calculated using data on the average amount of food consumed by the target group per day and the average concentration of dye used in the food formula (2). The data were then compared to the maximum ADI of each food dye (World Health Organization, 2017). These comparisons were divided into three categories: Scenarios A (values with average levels of detected colors and average consumption of foods); Scenario B (values with 95th percentile levels of detected colors and average consumption of foods); and Scenario C (values with 95th percentile levels of detected colors and 95th percentile consumption of foods) (Sumita Dixit et al., 2011).

Formula 2:

CDI (mg kg/bw) = Concentration of color present in food (mg/kg) × amount of colored food consumed (g or ml)/body weight (kg) (S Dixit et al., 2010).

### 2.6 Risk assessment of synthetic food dyes

The target hazard quotient (THQ) is the potential for non‐carcinogenic health effects caused by synthetic food dyes, which is calculated using the following formula (3):

Formula 3:

THQ = CDI/ RFD.

RFD is the reference dose (mg/kg bw/day). HI (Hazard Index) is the sum of all THQs in food samples. When THQ and HI are greater than one, a non‐carcinogenic risk is likely to occur (Pinto et al., 2020; Zahedi et al., 2020).

### 2.7 Statistical analysis

For data analysis, SPSS software version 16 was used. Analysis of variance (ANOVA) and the Independent‐Sample *t*‐test were used to compare the ADI of the desired synthetic dyes via consumption of four food items.

## 3 RESULTS

The results of analyzing food samples collected from Shiraz supermarkets and the types of dyes used in samples are summarized in Table 2.

**TABLE 2:** Permitted and non‐permitted colors in of 4 types of food products

		With permitted colors of sunset yellow and quinoline yellow			With non‐ permitted colors of tartrazine
Type of food	Total samples (n)	Total (%)	Within the standard (%)	Above the standard (%)	Total (%)
Ice product	25	22(88)	18(81.82)	4(18.18)	22(88)
Dairy‐free fruit ice cream	20	1(0.5)	—	1(100)	10(5)
Jelly	57	46(80.7)	45(97.83)	1(2.17)	48(84.21)
Candy	48	38(79.17)	37(97.37)	1(2.63)	33(68.75)
Total	150	107(71.33)	100(93.46)	7(6.54)	113(75.33)

According to the data, of 150 food samples, an average of 71.33% contained sunset yellow and quinoline yellow; and all these samples contained a mixture of tartrazine as well. Moreover, the use of authorized dyes in 6.54% of samples exceeded the standard level and 7.33% of samples contained only tartrazine. For jelly and candy products, the Codex standard was used, while other products were measured by Iran National Standards Organization Standard (INSO) No. 3964 (INSO, 2018; World Health Organization, 2011).

Table 3 summarizes the average consumption of quinoline yellow and sunset yellow dyes, as well as the tartrazine in each product. As the results indicate, the highest levels of tartrazine were found in candy and jelly products. Quinoline yellow was found in the highest concentrations in jelly, candy, and freeze pop, respectively. Sunset yellow dye was found in the highest concentrations in candy, freeze pop, and jelly, respectively. Finally, fruit ice creams contained the lowest concentration of sunset yellow. Overall, sunset yellow had the highest mean synthetic dye concentration in the samples (21.83 mg/kg).

**TABLE 3:** Average of the artificial colors of sunset yellow, quinoline yellow, and tartrazine in each product

Type of food	Tartrazine Mean ± SE (mg/kg or mg/l)	Quinoline yellow Mean ± SE (mg/kg or mg/l)	Sunset yellow Mean ± SE (mg/kg or mg/l)
Ice product	0.46 (±0.11)	11.76 (±3.89)	28.61 (±4.48)
Dairy‐free fruit ice cream	0.26 (±0.06)	0.00 (±0.00)	0.37 (±0.37)
Jelly	2.30 (±1.18)	17.96 (±3.32)	16.73 (±3.49)
Candy	33.61 (±16.08)	12.87 (±4.69)	35.65 (±8.20)
Average	10.96 (±4.92)	12.58 (±2.03)	21.83 (±3.04)

**FIGURE 1:**
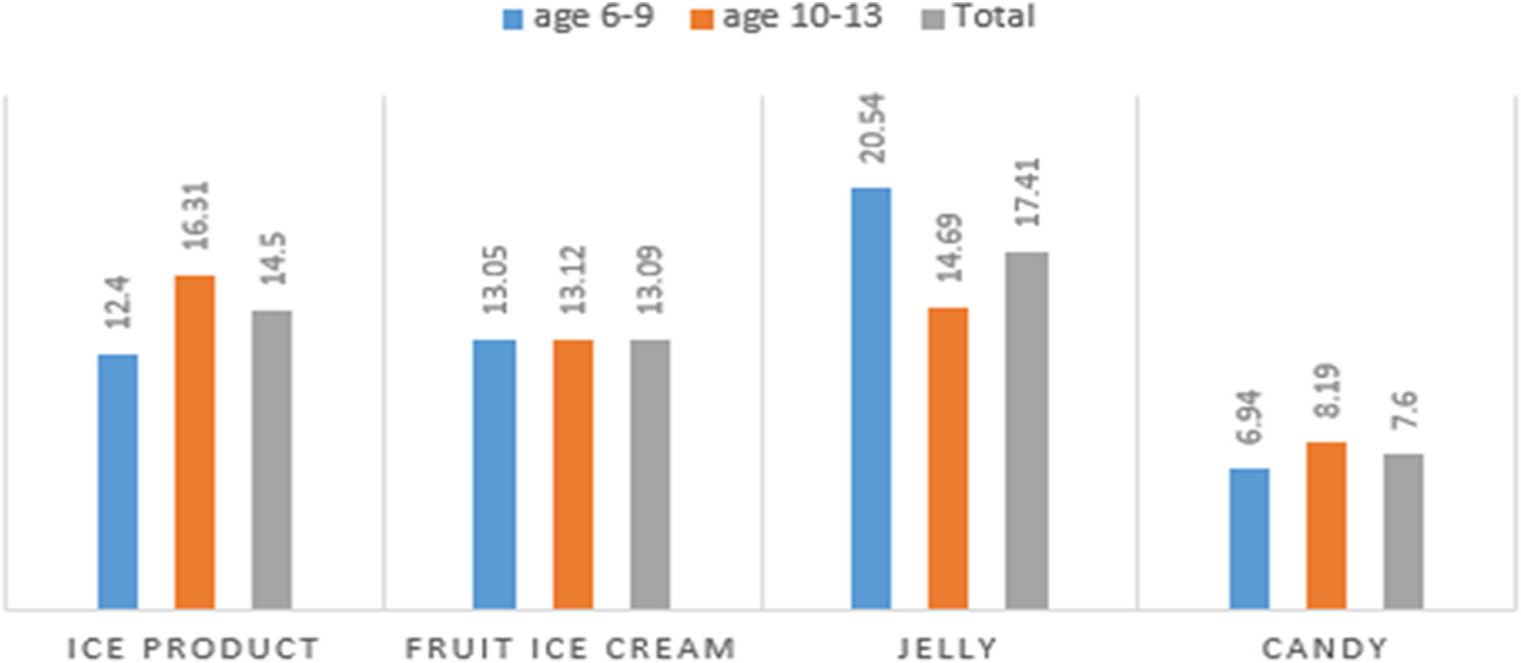
Consumption of foods containing artificial colors based on the type of product consumed daily in the two agegroups of 6–9 years and 13–10 years.

The most frequently consumed food containing dye by children was jelly (17.41 g/day), followed by freeze pop (14.50 g/day). Candy was the least consumed food dye on average (7.6 g/day). Jelly was the most consumed food dye among children aged 6–9 years, while the lowest average consumption was seen in candies. However, in children aged 10–13 years, the highest and lowest average consumption rates were related to freeze pop and candies, respectively (Figure 1).

**FIGURE 2:**
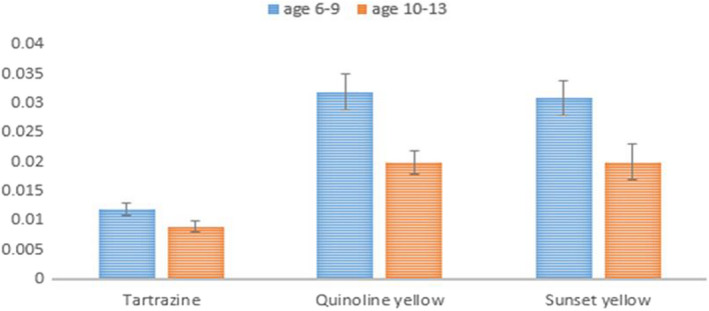
Intake (mean ± SD) of tartrazine, quinoline yellow and sunset yellow in the two age groups of children

The results indicated a statistically significant difference in the consumption of all three dyes between the two age groups, regardless of gender (*P* < 05.0). Also, the age group of 6–9 years old consumed more colored foods than the age group of 10–13 years old (Figure 2).

Scenarios A, B, and C were used to assess food dye consumption in the two age groups (Table 4). Comparing the values obtained for each color in the study revealed that the exposure rate was lower than ADIs in all three scenarios for both age groups.

**TABLE 4:** Exposure assessment of food colors in the two age group

			Intake of colors and ADI saturation (%)			
			6–9 years		10–13 years	
	Food colors	ADI (mg/kgbw)	Intake (mg kg/ bwt day)	Percentage ADI saturation	Intake (mg kg/ bwt day)	Percentage ADI saturation
Scenario A	Sunset yellow Quinoline yellow	0–4 0–5	0.03 0.03	0.78 0.64	0.02 0.01	0.50 0.38
Scenario B	Sunset yellow Quinoline yellow	0–4 0–5	0.23 0.25	5.75 5.00	0.09 0.11	2.25 2.20
Scenario C	Sunset yellow Quinoline yellow	0–4 0–5	0.70 0.74	17.50 14.80	0.32 0.42	8.00 8.40

*Note*: Scenario A: Values with average levels of detected colors and average consumption of foods. Scenario B: Values with 95th percentile levels of detected colors and average consumption of foods. Scenario C: Values with 95th levels of detected colors and 95th consumption of foods.

The HIs for quinoline yellow and sunset yellow in the food samples are presented separately for each age group in Tables 5 and 6. THQ and HI values were less than 1 in both age groups.

**TABLE 5:** Non‐carcinogenic health risks associated with ingestion of sunset yellow and quinoline yellow in various foods in children aged 6–9 years old

Type of food	Sunset yellow		Quinoline yellow	
	^a^CDI (±SE) (mg/kgbw/day)	^b^THQ	CDI(± SE) (mg/kgbw/day)	THQ
Dairy‐free fruit ice cream	0.00031(±0.00004)	0.00008	0.00000	0.00000
Ice product	0.01110 (±0.00176)	0.00277	0.01050 (±0.00179)	0.00210
Jelly	0.01189 (±0.00126)	0.00297	0.01654 (±0.00174)	0.00330
Candy	0.00783 (±0.00088)	0.00196	0.00458 (±0.00046)	0.00091
Total	0.03113	^c^HI = 0.00778	0.03162	HI = 0.00632

^a^ CDI Chronic daily intake (The average concentration of each synthetic dyes was taken as the residue level).

^b^ THQ target hazard quotient.

^c^ HI Hazard index.

**TABLE 6:** Non‐carcinogenic health risks associated with ingestion of sunset yellow and quinoline yellow in various foods in children aged 10–13 years old

Type of food	Sunset yellow		Quinoline yellow	
	^a^CDI (±SE) (mg/kgbw/day)	^b^THQ	CDI (±SE) (mg/kgbw/day)	THQ
Dairy‐free fruit ice cream	0.00019 ± 0.00002	0.00004	0.00000	0.00000
Ice product	0.00847 ± 0.00124	0.00211	0.00956 ± 0.00146	0.00191
Jelly	0.00530 ± 0.00063	0.00132	0.00746 ± 0.00084	0.00149
Candy	0.00617 ± 0.00061	0.00154	0.00286 ± 0.00027	0.00057
Total	0.02013	^c^HI = 0.0050	0.01988	HI = 0.00397

^a^ CDI Chronic daily intake (The average concentration of each synthetic dyes was taken as the residue level).

^b^ THQ target hazard quotient.

^c^ HI Hazard index.

## 4 DISCUSSION

In this study, we measured the amounts of permitted synthetic dyes, including quinoline yellow and sunset yellow, and evaluated the use of unauthorized tartrazine dye in four food products for children. Our results showed that 33.71% of colored foods contained both authorized and unauthorized food dyes. Moreover, tartrazine was found in samples, which is an unauthorized dye as defined by INSO No. 740. However, countries such as China, Japan, Australia, the United States, Europe, and India have legalized using this dye. In India, tartrazine is a widely used food dye (Tripathi et al., 2007; Yamjala et al., 2016).

Generally, the permitted amount of synthetic food dyes was within the standard limit in most samples. Only 6.54% of food samples were found to contain higher concentrations of sunset yellow and quinoline yellow dyes. The Codex standard was used for jelly and candy samples, and for comparing other products INSO No. 3964 was used (20). These results are close to those reported by Ahmed Asif Mohammed (2020) in Saudi Arabia and Ha. S.M et al. (2013) in South Korea (Ahmed et al., 2021; Ha et al., 2013).

Additionally, the results indicated that sunset yellow was the most frequently used dye in the samples, with an average concentration of 21.8329 mg/kg. Meanwhile, tartrazine was the least frequently used dye with an average concentration of 10.9640 mg/kg. Although the use of tartrazine is prohibited in Iran, it was found in almost all food products, particularly in 50% of ice cream samples. According to the INSO No. 2450, the use of any synthetic food dye in ice cream is prohibited (INSO, 2020). In most products, the concentration of this unauthorized color additive was less than 1 ppm. According to Hee‐Jae Suh et al. (2012), tartrazine was the most frequently used food dye in South Korea followed by brilliant blue, allura red, and sunset yellow. Furthermore, synthetic dyes were mostly used in candies and chocolates, and sunset yellow was the most used dye in candies (with high concentrations). These results were almost similar to our results. Zahedi et al. (2020) conducted a similar study in Kashan, Iran and evaluated 74 food samples including jellies, freeze pops, jelly powder, fruit snacks, and beverages in terms of children's exposure to synthetic dyes. The results showed that sunset yellow was the most commonly used dye (45%) in samples (Zahedi et al., 2020). In the present study, while tartrazine was the most commonly used unauthorized dye in food products, sunset yellow was the most commonly used authorized dye. Alves et al. (2008) studied synthetic dyes in Brazilian food products and concluded that sunset yellow was the most widely used food dye (Alves et al., 2008). The results were consistent with the results of this study. According to their results, allura red and sunset yellow were the most widely used dyes, and the lowest concentration of dye in foods was related to tartrazine followed by green dye (Hasan et al., 2018).

According to the findings of this study, jelly was the most popular dye among children aged 6–9 years, while freeze pops were the most popular food among children aged 10–13 years. Although candies contained the highest concentration of synthetic dyes, the consumption was low in both age groups. So, children were exposed to synthetic dyes mainly through the consumption of jellies and freeze pops. Sumita Dixit et al. (2011) in India showed that beverages and icy candies were the most consumed foods, which was inconsistent with our results. Also, contrary to our results, A. Husain et al. (2006) in Kuwait showed that children consumed beverages and juices more than other foods (Sumita Dixit et al., 2011; Husain et al., 2006). However, it should be noted that the mentioned studies evaluated a broad range of food products, whereas the current study examined only a limited number of food products due to study limitations.

Although some dyes were used in higher concentrations than the standard levels, the results indicated that intake of quinoline yellow and sunset yellow was lower than ADIs in both groups at three levels of medium (Scenario A), 95th percentile (Scenario B), and (Scenario C) (EFSA, 2009). Additionally, the non‐carcinogenic risks associated with these dyes were evaluated, and the THQ and HI were estimated to be less than 1, indicating that exposure to these two dyes through the consumption of dairy‐free fruit ice cream, freeze pop, jelly, and candy did not pose a risk to children's general health. The results of the studies by Zahedi et al. (2020) in Iran, Ha. S.‐M et al. (2013) in South Korea, and Hee‐Jae Suh et al. (2012) in South Korea were consistent with our findings (Ha et al., 2013; Suh & Choi, 2012; Zahedi et al., 2020). Contrary to our findings, the results of the studies by S. Dixit et al. (2010 and 2011) in India and A. Husain et al. (2006) in Kuwait showed that, at the moderate level of scenario (A), the consumption of certain dyes, particularly sunset yellow and tartrazine, exceeded the respective ADI (S Dixit et al., 2010; Sumita Dixit et al., 2011; Husain et al., 2006).

According to the current research, children's consumption of the two authorized dyes was lower than the ADI, and there was a low risk of health problems among the children in this study. However, it should be noted that we studied only four types of products containing color additives; the results would likely be very different if other colors were included. Considering that several previous studies reported unhealthy effects of synthetic dyes among children (e.g., exacerbating hyperactivity symptoms), the use of these dyes should be monitored carefully.

This study had several limitations. First, the samples of this study were from a single group with a close socioeconomic status (the SUMS staff's children), which restricts the generalization of our results to other populations. Second, due to the COVID‐19 pandemic, a relatively small sample size was selected from the available population. Third, while there were some other products containing artificial colors, we only considered four types of food products due to feasibility of the study.

Epidemics may also change people's perceptions of healthy eating and greatly reduce the consumption of colored foods (Janssen et al., 2021). In this study, the total concentration of synthetic dyes in food samples was analyzed, which may lead to overestimation of health risks. Therefore, in order to accurately estimate the health risks, an appropriate form of artificial colors must be selected. However, our study was the first to evaluate the risk assessment of three synthetic dyes in four food products in Shiraz, Iran.

## 5 CONCLUSION

In this study, the level of risk to children's health posed by the authorized synthetic dyes was deemed acceptable. Thus, the studied population can safely consume the above‐mentioned foods in Shiraz. Although tartrazine is an unauthorized synthetic dye in Iran, it was used in most food products evaluated in the current study, and most remarkably in ice cream. Regarding the potential dangers associated with the consumption of high concentrations of authorized and unauthorized synthetic dyes to health, particularly in children, continuous monitoring is necessary to maintain control over these dyes. Further research is required to obtain more valid results by including additional food products, expanding the target group, and considering more synthetic dyes.
